# Facilitators of Blood Pressure Control Among Treated Patients in Public Clinics in Botswana

**DOI:** 10.1002/puh2.70328

**Published:** 2026-08-07

**Authors:** Vincent Setlhare, Yaone Bogatsu, Nabila Youssouf, Julius C Mwita

**Affiliations:** ^1^ Department of Family Medicine and Primary Healthcare University of Botswana Gaborone Botswana; ^2^ Department of Clinical Research London School of Hygiene and Tropical Medicine London UK; ^3^ Department of Internal Medicine University of Botswana Gaborone Botswana

**Keywords:** facilitators, improvers, primary healthcare (PHC) clinics, uncontrolled blood pressure (BP)

## Abstract

**Background:**

High blood pressure (BP) prevalence is increasing in low‐ and middle‐income countries, leading to increased morbidity and mortality. This compromises Sustainable Development Goal number 3—to ensure healthy lives for all people. Most BP patients do not attain normal BP despite treatment. Uncontrolled BP causes cardiovascular diseases, kidney disease and early death. It is therefore prudent to know the facilitators of BP control among treated patients.

**Methods:**

The aim of this study was to explore the facilitators of BP control among treated patients in two public primary healthcare (PHC) clinics in Gaborone, Botswana. Participants were purposively selected to ensure the selection of participants with good knowledge of high BP. These were patients with more than 1 year experience of BP treatment, healthcare workers (HCWs) with more than 3 years’ experience of treating BP patients, and district health managers. Semi‐structured interviews were used to collect data until saturation, from BP patients, nurses, doctors and district health managers. Interviews were audio recorded, transcribed and then coded by three authors. The codes were iteratively grouped and regrouped into themes several times to produce a codebook. The codebook and transcripts were uploaded into NVivo for analysing all the transcripts.

**Results:**

Eighteen participants (six patients, four nurses, four doctors and four district health managers) were interviewed. The themes that were extracted from the data included health system, HCW, patient and socio‐economic related factors.

**Conclusion:**

The perceived facilitators of BP control among patients receiving BP treatment included central government, local government and public PHC clinic level factors. Perceived facilitating factors also included patient, and HCW components. The study findings present a firm base from which more extensive qualitative and quantitative studies can be made to confirm or disprove the generalisability of these findings to all the public PHC clinics in Botswana.

## Graphical abstract

Most (63%) of the high BP patients in Botswana do not reach normal BP despite treatment in public primary healthcare clinics. This may result in illness and death. Our study findings were that health system, clinic management, healthcare worker, patient and patient support factors could possibly improve BP control rates.

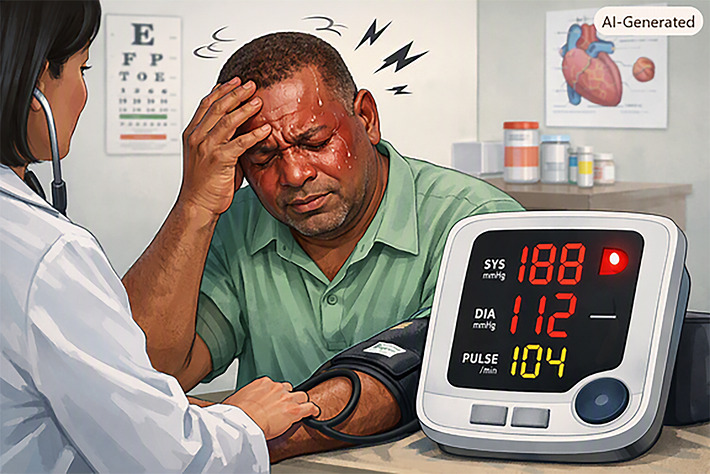

## 1 Introduction

Sustainable Development Goal number 3 (SDG 3) aspires to ensure healthy lives and promoting the well‐being of all at all ages. One of the biggest challenges to SDG 3 is the increasing morbidity and mortality due to cardiovascular diseases (CVDs), the leading cause of death worldwide [1, 2]. Most (38%) of premature deaths (17.9 million) in 2019 were caused by CVDs [2]. In 2021, 20.5 million people died of CVDs, and it is estimated that 80% of these deaths occurred in low‐ and middle‐income countries (LMICs), at a young age [3, 4]. This is because LMICs have weak health systems and resource constraints [5, 6, 7]. The control of high blood pressure (BP) can contribute to reducing CVD morbidity and mortality, worldwide.

Between 1990 and 2019, there was a 41% increase in people with high BP in Europe and the Americas, whereas South‐East Asia and the Western Pacific region experienced a 144% increase [8]. In sub‐Saharan Africa, the increase was 48% and 34% in women and men, respectively [9].

High BP is the springboard of CVD morbidity and mortality as already stated [10]. Over one billion (1.13–1.39 billion) people worldwide have high BP despite the availability of preventive and curative interventions [4, 11]. The prevalence of high BP is highest in LMICs [4], and sub‐Saharan Africa carries the highest burden [12].

The control of high BP is challenging in both high income countries (HICs) and LMICs [13, 14, 15]. BP control rates in treated patients range from >50% in HICs to as low as 10% in some LMICs [16]. Worldwide, only 21% of people suffering from high BP have their BP under control [14]. Increasing the percentage of patients with controlled BP can decrease CVD morbidity and mortality, and the attendant costs of care, and burial.

Success in BP control depends on implementing evidence‐based, context‐specific strategies of BP control among treated patients [15]. Translating evidence‐based interventions into successful practice is not simple and requires a good knowledge of implementation research and practice [17].

Facilitators of BP control include healthy diets, exercising regularly, detecting high BP early and taking effective treatment that is prescribed correctly [14]. Strategies to improve BP control among adult patients in the United States included attention to racial issues, measuring BP accurately, self‐monitoring of BP, standardising care, using clinical teams to administer BP care, improving adherence to medication and promoting lifestyle change [13]. To lower CVD morbidity and mortality, sub‐Saharan Africa countries need to adopt the WHO HEARTS protocols, that is, adopting healthy diets, implementing effective BP screening and treatment interventions, combating physician treatment inertia, improving adherence to treatment, increasing access to care, and equipping healthcare workers (HCWs) with the necessary skills and knowledge [18].

Botswana is one of the sub‐Saharan countries with low BP control rates among treated patients, with 63% of treated patients living with uncontrolled BP (BP regularly ≥140/90) [19]. The aim of this study was to explore the facilitators of BP control among patients attending two public primary healthcare (PHC) clinics in Gaborone, Botswana.

In the context of this study, the term ‘facilitators’ includes all factors that participants thought were enablers of better control of high BP. We hoped that the study findings would help one to inform strategies to improve BP control among patients receiving treatment in public PHC clinics.

We chose a descriptive qualitative approach to study the facilitators of BP control because this methodology is suitable to study complex phenomena (e.g., lack of BP control despite knowledge of evidence‐based interventions) that affect health [20]. Some would argue that qualitative studies are the best method for studying barriers and facilitators of public health interventions [21]. In 2021, ischaemic heart disease and stroke were the first and third (respectively) highest causes of mortality worldwide and most of these deaths were in LMICs [22]. Thus, this study was guided by the choice of an appropriate methodology to study a complex issue, and by the urgency to control a world‐wide disease that is causing high morbidity and mortality.

## 2 Methods

This was a descriptive qualitative study [23] that explored the facilitators of BP control among treated patients in two public PHC clinics in Gaborone, Botswana. The two public PHC clinics are situated at opposite ends of the city and serve high numbers of patients daily. These patients (adults and children, men and women) present with the full spectrum of diseases ranging from infectious and non‐communicable diseases to obstetric and gynaecological problems, to children's diseases, to trauma and surgical problems. Nurses and doctors consult with these patients, diagnose their problems and give them treatment.

Purposive sampling was used to select suitable participants (patients, nurses, doctors and district health managers) who varied in age, gender and BP experience. Patients who were on treatment for high BP, as well as nurses and doctors who consulted these patients, were selected face to face, from two, high patient volume, public PHC clinics in Gaborone, Botswana. District health managers were selected face to face from the Gaborone District Health Management Team offices. Data were collected through semi‐structured, audio recorded interviews, using interview guides.

Data were collected by a research assistant (RA) who was trained in qualitative research, and in interviewing techniques. Among the authors, V.S. was a male family physician (MFamMed) who had many years of experience working in public PHC clinics, treating BP patients and teaching family medicine at undergraduate and at graduate level. V.S. has many publications using qualitative research methods. Y.B. was a female family physician (MMed) with many years of experience working in public PHC clinics. She also teaches family medicine at undergraduate and graduate level. N.Y. was a female clinical project manager with many years of experience in managing UK and international research projects. She is a geneticist (PhD) by training. J.M. was a male cardiologist (MMed) with several publications on cardiovascular topics and has worked in public PHC clinics for years.

As clinicians, V.S., Y.B. and J.M. were familiar with the Botswana public PHC clinics and the type of patients getting BP treatment there. Participants in this study did not know the identity of the researchers involved in this study because the RA was told to hide their identity, to avoid participant bias.

Patient participants and nurse participants were interviewed in privacy, in a room, at the public PHC clinics. Doctor participants were interviewed in privacy at the clinics, or at a quiet secluded venue of their choice, outside the clinics. District health managers were interviewed at their offices, in privacy.

An interview guide was crafted from the literature and used to collect data from participants. The interview guide was developed further during the initial five interviews to make it suitable for the study. The researcher (V.S.) and the RA listened to the first five interviews together to identify any problems in interviewing technique, or ease of application of the interview guide, or comprehension of the interview guide. Any problems in this regard were discussed, and a satisfactory solution was agreed upon. This helped develop the interview guide and the interviewing technique to optimise data collection.

The interview guide was semi‐structured and it directed participants to express their views on (i) reasons why BP remained high among treated BP patients, (ii) what could be done to improve BP control, (iii) issues concerning medical drugs or medical equipment that affected BP control, (iv) clinic management factors that affected BP control, and (v) behaviours and attitudes of patients and HCWs that affected BP control. Probes, follow‐up questions and requests for explanations were used to collect deep data. Interviews were conducted in the local language with most patients. Nurses, doctors and health managers were interviewed in English, or the local language, or both. Field notes were taken during data collection, and they described RA observations about participants, the environment of the interview and the data. There were two repeat interviews, to complete interviews that had not been concluded. The interviews were audio recorded and then the audio‐recordings were uploaded into a password‐locked laptop.

In Botswana public PHC clinics, the human determinants of BP control are mainly centred around the interactions of patients, HCWs (nurses and doctors who consult with patients) and district health managers who oversee public PHC clinics. These stakeholders in BP control were interviewed as one group, who had a good understanding of BP control in public PHC clinics. To get deep data and to minimise wastage of resources, qualitative researchers often use participants with good knowledge of the subject of the study [24, 25]. We conducted interviews until data saturation [26].

Data saturation is a topic of debate in qualitative research [27, 28]. Data saturation can be described as theoretical saturation, code saturation or thematic saturation (inductive or a priori), data saturation and meaning saturation to suit different methods of qualitative research. We used the data saturation concept to indicate a point in data collection, when new interviews were increasingly yielding redundant data to the point that it was not worthwhile to continue collecting data.

V.S. listened to each audio‐taped recording as it was delivered to him by the RA. V.S. discussed the essence of what was being said by participants (data), with the RA. There came a point when V.S. and the RA agreed that further interviews were increasingly yielding very little new data. It is at this point that data saturation was thought to be reached [28, 29]. As we wanted to get a balanced understanding of the problem, we ensured that we had a fairly equal representation of each group of participants at saturation. Our sample size, at data saturation, exceeded the expected number [30].

The interview audio‐recordings were then transcribed. The Setswana interviews were transcribed into Setswana by the RA, and then V.S. randomly chose five Setswana transcripts and read each transcript while listening to its audio recording. V.S. found the transcripts to be good transcriptions of the Setswana audio‐recordings. The Setswana transcripts were translated into English by the RA. V.S. read four random English translations of the Setswana transcripts, comparing them to the Setswana transcripts. He found them to be good translations. The English audio‐recorded interviews were similarly transcribed by the RA and checked by V.S. for accuracy. V.S. found them to be good transcripts of the English audio‐recorded interviews. Both the RA and the researcher had good command of both the local language (Setswana) and English. Moreover, they were both familiar with the Botswana public PHC clinics, and the types of patients and health workers found in these clinics.

The analysis of the data was done from a realist, intuitive, semantic approach [31]. We crafted codes and themes from the whole data set such that the themes and subthemes represented what was in the data, with minimal overinterpretation or theorising about the data [23, 32]. The data were analysed for themes using the method described by Braun and Clarke [31]. Three authors (V.S., Y.B. and N.Y.) read and reread the transcripts several times to familiarise themselves with the data. Then they independently coded the same three transcripts. Thereafter, they discussed their codes and agreed on it to be used for coding. V.S. then grouped and regrouped the codes several times until he crafted a codebook. The codebook was subjected to iterative discussions among V.S., Y.B. and N.Y. until consensus was reached.

The codebook and the English transcripts were uploaded into NVivo 14. All the English transcripts were then analysed using NVivo 14, by V.S. After the dataset was analysed, V.S., Y.B. and N.Y. revisited the themes and iteratively regrouped them, broke them down, crafted new themes, deleted themes and added new themes until they were satisfied with the themes they had made from the data.

Y.B. and V.S. are both family physicians and they are influenced by and practitioners of the *biopsychosocial approach* to medicine. This approach is grounded in the view that human health is influenced by biological, psychological and social phenomena. Both V.S. and Y.B. reminded themselves that their analyses were likely to be biased towards biological, psychological and social factors. However, N.Y. did not have this bias because of her training (master's and PhD degrees in Human Molecular Genetics, and Statistical Genetics respectively) which likely made her analyses to be biased towards the biological. Author J.M. (a cardiologist) is a quantitative researcher and would have been more biased towards the clinical facilitators of BP control, when he reviewed the manuscript. During the analyses of the data and reviews of the manuscript we challenged each other to be aware of our likely biases, and hopefully cancelled them out because of our different backgrounds.

### 2.1 Ethical Considerations

Participants were interviewed after the study was thoroughly explained to them and after their questions about the study were answered to their satisfaction. The RA advised participants that they had the right to stop participating in the study at any time, if they so wished. The RA assured the participants that there was minimal risk in participating in the research and gave them the contact numbers of persons to contact in the remote chance that they were adversely affected by the study. A small token of appreciation was given to participants at the end of each interview.

Only consenting adults; BP patients, nurses and doctors who consulted patients in the two public PHC study clinics, as well as district health managers who supervised these clinics, participated in the study. Participants showed their consent to participate in the study clinics by putting their signatures on the consent forms (one being consent to participate and the other being consent for the interview to be audio recorded). Those who could not write put a cross (X) against their name as proof of consent.

## 3 Results

Eighteen adult participants consisting of 13 females, 6 patients, 4 nurses, 4 doctors and 4 district health managers were interviewed (Table 1). Very few participants refused to participate, and those who did, did so because of time constrains. Participants’ ages ranged from 27 to 60 years. The participants were recruited from two high patient volumes, 24‐h clinics with maternity wings, on opposite sides of Gaborone, Botswana.

**TABLE 1:** Participants’ demographic information.

S/No.	Participant number	Facility	Sex	Age	Employment status	Designation	Education level
1	14	DHMT	F	45	Employed	Head—EMS	Tertiary
2	16	DHMT	F	Not provided	Employed	Head—Pharmacy	Tertiary
3	17	DHMT	M	Not provided	Employed	Head—Curative Medicine	Tertiary
4	11	DHMT	F	56	Employed	Head—Nursing	Tertiary
5	18	Juliah Molefhe	M	27	Employed	Medical Officer	Tertiary
6	20	Juliah Molefhe	F	37	Employed	Medical Officer	Tertiary
7	07	Juliah Molefhe	F	33	Employed	Medical Officer	Tertiary
8	19	Juliah Molefhe	M	34	Employed	Medical Officer	Tertiary
9	03	Juliah Molefhe	M	33	Employed	Nurse	Tertiary
10	05	Juliah Molefhe	F	25	Employed	Nurse	Tertiary
11	08	Broadhurst III	F	42	Employed	Nurse	Tertiary
12	13	Broadhurst III	F	39	Employed	Nurse	Tertiary
13	01	Juliah Molefhe	F	43	Unemployed	Patient	Secondary
14	02	Juliah Molefhe	F	50	Employed	Patient	Secondary
15	04	Juliah Molefhe	F	60	Employed	Patient	Secondary
16	06	Juliah Molefhe	M	27	Employed	Patient	Tertiary
17	09	Broadhurst III	F	40	Self‐employed	Patient	Secondary
18	10	Broadhurst III	F	58	Unemployed	Patient	Primary

Abbreviation: EMS, emergency medical services.

The shortest interview lasted for 17 min 33 s, whereas the longest was for 46 min 54 s. The average time for each interview was 33 min 20 s.

At the initial analysis of the data, the three authors (Y.B., N.Y. and V.S.) independently coded the same three transcripts. They then discussed their codes and greed on the codes to be used to analyse the data. After V.S. analysed the dataset, the three authors grouped and regrouped the codes into 177 codes and 15 themes (Table 2).

**TABLE 2:** First draft of themes and codes.

Themes	Codes (recurring words, phrases, ideas)
Staff patient relations	Treating patients nicely, discussions with patients, counselling, how patient feels about medication, referrals to social worker, dietician
BP machines	Calibration and recalibration BP machines, expertise of those using BP machines
BP control	Lifestyle, adherence, BP readings, knowledge of drugs, importance of drugs, spending time with patient, need for someone to talk to, space to talk, patient seen by the same doctor, need for team work
BP clinic	Registration of BP patients, keeping track of statistics
Referrals	To dietician, social worker
Patient review/monitoring	Checking BP, teach, check for social problems
Adherence	Care giver role, taking medications at right time, taking medications everyday
Lifestyle issues	Diet, exercise, employment
Patient education	Counselling, teaching, morning sessions, sometimes done daily, information, dietician
Treatment	Treatment guidelines
What brings BP down	BP clinic for BP patients, taking treatment, change of treatment, adherence
Exercise	Taking a walk
Food	Eating BP related food
Self‐care	Self‐encouragement, self‐discipline, self‐motivation, patients should take care of themselves
BP medicines	Government responsibility, increase supply of pills, free supply from pharmacies

Abbreviation: BP, blood pressure.

The three authors agreed that these 15 themes and 177 codes could be regrouped into fewer codes and themes. Author V.S. was tasked with doing this and to present an updated draft to Y.B. and N.Y. The three authors met after a week to discuss the updated draft that V.S. presented. After several discussions, the three authors reached a consensus to reduce the number of themes to 7, as shown in Table 3.

**TABLE 3:** Second draft of themes and codes.

**BP control**: Controlled BP, what brings BP down, normal BP, taking treatment, change of treatment, nurse consultations
**Clinic processes**: Need for BP clinic, controlled patients seldom consult with doctors
**Life style issues**: Exercise, correct diet; home cooking, social clubs
**Personal responsibility**: Self‐encouragement, self‐discipline, self‐motivation, going to clinic early, self‐care, herbs
**Information/Communication issues**: Registering for free drug supply in pharmacies
**Government responsibility**: Pills/Medications; increase supply of pills, free supply from pharmacies, increase staff, don't leave everything to patients (government neglecting it's duty), Leadership/administration to enforce laws BP machines, expertise of those using BP machines, personal BP machines
**Doctors**: Doctor consultations, sometimes helpful, change medication, use BP guidelines, attitudes/behaviours

Abbreviation: BP, blood pressure.

The authors still felt the need to make the findings clearer and concise. V.S. further grouped and regrouped the second draft of themes and codes and presented them to Y.B. and N.Y. for discussion. Several discussions later, consensus was reached on the final themes and codes as represented in Table 4.

**TABLE 4:** Final draft of themes and codes.

**Patient related**—Lifestyle, self‐care, adherence, individual contribution
**Health system related**—Medicine supply, BP machines, BP cuffs, BP clinic, waiting time, digital health records, increase staff, continuity of care, combination pills
**HCW related—**Change medications, adherence counselling, reduce pill burden, health promotion and education, BP management, pharmacy to check prescriptions, BP check before re‐dispensing, evaluate co‐morbidities, use guidelines
**Social support—**Family, social, economic

Abbreviations: BP, blood pressure; HCW, healthcare worker.

On thematic analysis of the data, the perceived facilitators of BP control among treated patients in the two study clinics were categorised into the following final themes: healthcare system related, HCW related, patient related and social support related.

### 3.1 The Health System–Related Facilitators of BP Control

The perceived facilitators of BP control among treated patients included, having a specialised BP clinic, having doctors dedicated to the BP clinic, having more doctors, ensuring that BP medicines are always available, outsourcing of BP medicines, using electronic BP machines and having a clinical team to treat BP patients.

Participant 12 (a patient) said, ‘There can be doctors who are specifically focused on BP; there …’ ‘there should be a BP clinic because it looks like BP patients are now too many’.

‘It's just to have medication in the hospital (clinic) because if there are no medicines there is no situation that can improve’, said Participant 09 (a patient).

‘… government is willing to pay somebody … to have them registered at a private pharmacy to be paid (the pharmacy to be paid by government) for when they (patients) have taken the medication (from the private pharmacy)’, said Participant 17 (district health manager, curative).

Participant 15 (a district health manager) said, ‘… its just that if it could be possible for them (patients) to be booked for dieticians (for patients to be referred to dieticians) to monitor all these (dietary) things that are surrounding … or what is aggravating the problem you see?’.

### 3.2 HCW‐Related Perceived Facilitators of BP Control

These included having a good knowledge of BP treatment, counselling patients on good lifestyle habits and counselling patients about adherence to medication. Clinicians also needed to have a good attitude and consult each other about uncontrollable BP cases.

Participant 18 (a medical doctor) thought, ‘Standardization of treatment is important and the only way that can be done is if all the doctors are educated about the same thing (same way of BP treatment)’.

A district health manager (Participant 16) said, ‘(when you wander) what is happening to this patient, what are the issues (of his uncontrolled BP)? And those issues you share them (discuss them) with other helpers (HCWs)’.

Participant 09 (a patient) wished that HCWs should talk nicely to patients. ‘Yes, he (the HCW) should talk to you well, being relaxed and you too being relaxed’.

Participant 04 (a patient) said, ‘I had multiple visits to the dietician. He then kept on teaching me how I should eat (the kind of meals one should eat)’.

‘We usually counsel them like when we have a newly diagnosed hypertensive, … like reduction of salt, frequent exercise, emmm … you know and all those aspects’, said Participant 07 (a doctor).

### 3.3 Patient‐Related Perceived Facilitators of BP Control

These included taking responsibility for BP care, adherence to BP treatment and adopting healthy lifestyles.

Participant 02 (a patient) said, ‘(A healthy) diet is the most important thing (for BP control) and exercising also’.

A nurse who was also a district health manager (Participant 15) said, ‘(it is the patient) who has BP, and it is his responsibility to come for review, it is his responsibility to eat well like I have already mentioned. It is his responsibility to take medication as prescribed’.

### 3.4 Social and Economic Support

Participants thought that social and economic support of BP patients, especially the elderly and poor, were facilitators of BP control.

‘This is how you cook for them (the elderly), less salt, less oil in their food. Make sure that at six o'clock every time, whatever time is suitable for them, in the morning they should take their medication at exactly that time frame’, said Participant 14, a nurse; illustrating the teaching given to caregivers, to support BP patients.

A district health manager (Participant 17) said, ‘And then if patients fail, (if) they are not showing up for medication, the family is called, and the patient is discussed. …. so it's more structured and it also involves the family and the patient's support system; from the family or from the relative or whoever they think will support them’.

‘A patient can be without money to go and see doctors but if the family is supportive, like the other one bringing a car, the other giving him two Pula (money) for transport, when he gets at the facility he would feel that “no there is hope in my sickness,” instead (of the elderly BP patient) just being there, hopeless, and without anyone around’, said a district health manager who was a nurse.

*The use of traditional medicine* was mentioned by one patient as a facilitator of BP control. Block 9 Patient 02 said, ‘Some people like a friend of mine said she stopped the (clinic) medication, but she is using these herbs … and the BP is okay, its normal’.

## 4 Discussion

The subthemes or the details that explain what each theme consisted of help one to appreciate the perceived facilitators of BP control in the two public PHC study clinics. Many of these subthemes describe facilitators that are currently not present in the Botswana public PHC clinics, for example, exclusive BP clinics, established clinical teams for discussing difficult BP cases and use of a structured BP patient social support system.

This study showed that participants thought that factors that facilitated BP control in the two study clinics were health system, HCW, patient and social support related. The subthemes explain what each theme highlighted.

The Botswana health system is challenged by HCW shortages resulting from increased healthcare demand, unequal rural and urban HCW staffing, inadequate production of HCWs, as well as internal and external migration of HCWs [5, 6].

This study corroborates studies that show that health systems need to be structured, resourced and run in such a way that they can deliver health services effectively [7, 14]. Our study showed that the health system should provide the study clinics with resources—uninterrupted supply of correct medications, functional BP equipment, a separate BP clinic and enough HCWs dedicated to a BP clinic—to facilitate BP control. Similar findings were documented in a study in Ghana, and a study in Nepal that showed the importance of the availability of adequate resources for BP control [33, 34]. The World Health Organization and the World BP League also advocate for adequate and appropriate resourcing of health systems, to control BP [14, 35]. A study in South Africa also highlighted the importance of the availability of adequate resources in the health system, to control BP [36]. Resource shortages in weak health systems therefore remain a challenge to BP control [37]. The lack of human and other healthcare resources in LMIC health systems remain a challenge to BP control in treated patients.

HCW‐related facilitators of BP control in the study clinics were thought to include knowledge of BP treatment, using a teamwork approach, having a positive attitude towards patients, and counselling patients on helpful lifestyles. The Botswana public PHC clinics are characterised by high patient volumes, and HCW shortages. High patient volumes do not permit enough time for counselling patients on the importance of diet and lifestyle, and they erode HCW attitudes and behaviours. Some studies have shown the adverse effects of high patient volumes on the quality of care [38, 39]. A study in poor communities in Senegal, however, showed that high patient volumes did not affect the quality of patient care [40]. This may have been due to high patient volumes being the exception rather than the norm, in the Senegal study.

Doctors’ knowledge of BP treatment is influenced by the user friendliness of BP guidelines, trust in the guideline, as well as the availability of equipment and medications [41]. Training HCWs to increase their knowledge of BP treatment improves BP control [42, 43]. The findings of our study affirm that counselling patients on beneficial lifestyles improves BP control [14]. However, adopting a healthy diet was not a significant factor of achieving BP control in one study in Lebanon [44]. This Lebanon study did not explain how following a healthy diet was monitored. This lapse may mean that following a healthy diet was not strictly monitored in that study, resulting in the finding that a healthy diet was not a significant factor in BP control. However, our findings that a team‐based treatment approach and lifestyle counselling facilitate BP control corroborate the scientific statement from the American Heart Association and the American Medical Association [13]. Our findings that positive doctor attitudes towards patients facilitate BP control align with the findings of a review article that showed that a good doctor–patient relationship improves BP control [45].

Patient‐related facilitators of BP control in our study clinics were believed to include taking responsibility for treatment, adherence to treatment and adopting healthy lifestyles. We thought that taking responsibility for treatment was an interesting subtheme which included patients ensuring that they kept their review appointments. Patients taking responsibility for their own treatment also overlapped with maintaining a healthy lifestyle and eating BP compatible foods. These patient‐related facilitators of BP control are in line with advice from WHO, the American Heart Association, the American Medical Association and the World BP League [13, 14, 35].

Social and economic supports of BP patients were mentioned as facilitators of BP control in our study clinics. The traditional extended family support for the elderly is eroding in Botswana. Many elderly people live alone, have challenges in reading doctors prescriptions and following them, and many cannot cook for themselves. This is why social support for the aged BP patient is necessary. Social support was a facilitator of BP control in a study in Nepal where family members changed their cooking practices and encouraged BP patients to adopt healthy diets [34].

Shortages of medicines in public clinics force patients to buy expensive medicines using their own money, and most patients need financial support to buy them. The need for economic support for low income BP patients is supported by a study which found increasing rates of uncontrolled BP as patients’ wealth decreased [46]. One study in Nigeria found that provision of health insurance tor BP patients improved BP control [47].

In this study, traditional medicine was perceived as a facilitator of BP control but a barrier to adherence to prescribed medicines. Many traditional medicines have been shown to decrease high BP but their superiority over modern BP drugs has not been demonstrated [48]. Mugisha of Uganda, in Africa, believed that traditional medicines can be used to enhance adherence to prescriptions [49]. However, a study in 12 sub‐Saharan African countries showed that traditional medicine use increased non‐adherence to medications, increased BP, increased severe BP and complications of BP [50]. These studies show that there is conflicting evidence in the utility of traditional medicine use in BP control.

The contribution of this study is that it highlights the perceived facilitators of BP control in two public PHC clinics in Botswana, thus paving a way to more extensive qualitative and quantitative studies to prove or disprove the representativeness of these findings across the Botswana public PHC clinics. These more extensive studies may help to inform strategies that can be used in Botswana to control BP among patients treated in public PHC clinics.

## 5 Conclusions

The findings of this qualitative study are only true for the two study clinics. The perceptions of participants were that BP control among treated patients may be improved by attending to health system, HCW, patient and socio‐economic support factors. The study tentatively indicates that a holistic systems approach needs to be crafted and adopted to improve BP control among treated patients in the study clinics. This holistic system approach should address health resource, HCW, patient and community level challenges.

### 5.1 Study Limitations

This was a qualitative study in two Gaborone public clinics. The results are therefore not necessarily generalisable although they corroborate findings from other studies and are in line with the views of respected international organisations that have expertise in BP control. This study could have benefitted from treating patients, nurses, doctors and health managers as different entities. Data would then have been collected until saturation, for each group. Separate data analysis for each group would have yielded clear perceptions of each group, thus making the findings richer. Involvement of privately owned public clinics may have influenced the results differently because such clinics are better resourced and treat patients who are generally better educated and well‐to‐do financially.

### 5.2 Recommendations

This is an important study that needs to be followed by more extensive qualitative and quantitative studies.

The study, though limited to two public PHC clinics in Gaborone, tentatively indicates that adequate health funding, procurement and management of enough resources, as well as training of HCWs and patients in BP control, should be prioritised.

## Author Contributions

**Vincent Setlhare**: conceptualisation, proposal and grant writing, analysis and manuscript writing. **Yaone Bogatsu**: analysis and manuscript writing. **Nabila Youssouf**: conceptualisation, analysis and manuscript writing. **Julius Mwita**: conceptualisation and manuscript writing.

## Funding

This study was funded by the National Institutes of Health (NIH), sub award no. WU‐23‐0456.

## Ethics Statement

This study received ethical approval from the University of Botswana (UBR/RES/IRB/BIO/343), from the Gaborone District Health Management Team (GGDHMT 6/17/1 III (39) and from the Ministry of Health (HPRD: 6/14/1).

## Consent

All participants gave written consent to participate in the study.

## Conflicts of Interest

The authors declare no conflicts of interest.

## Data Availability

The data underlying this article will be shared on reasonable request to the corresponding author.
